# Stoicism or Defeat? The Psychological Impact of the Kiln Environment on Working Donkeys and Mules

**DOI:** 10.3390/ani15111525

**Published:** 2025-05-23

**Authors:** Katy Taylor, Anna Harrison, Theodora Capaldo

**Affiliations:** 1 Independent Researcher, Sheffield, UK; katydtaylor@aol.com; 2 Independent Researcher, Exeter, UK; 3 American Fund for Alternatives to Animal Research, Georgetown, MA 01833, USA

**Keywords:** donkey, mules, brick kiln, welfare, depression, learned helplessness, PTSD

## Abstract

One of the most problematic situations for the estimated 60 million working donkeys and mules across the world is within the brick kiln environment. In countries such as Egypt, Nepal, India and Pakistan, donkeys and mules carry dried bricks into small kilns either by pulling carts or carrying loaded packs. The health and welfare problems of this environment are well documented: they may carry excessive loads; work long hours, have difficulties accessing food, water and rest options; and can frequently suffer untreated illnesses including dental problems, lameness and open wounds from poorly fitting harnesses and aggressive handlers. The impact of this environment on their mental health has been largely overlooked. We use a behavioral comparative analogy approach, comparing the clinical signs and risk factors associated with learned helplessness, depression and complex post-traumatic stress disorder (cPTSD) in humans and laboratory studies in other animals with evidence from the veterinary literature. Based on an analysis of seven quantitative veterinary studies that have evaluated the health and behavior of kiln donkeys and mules, we argue that their behavior may be consistent with learned helplessness, depression and/or cPTSD. Furthermore, the kiln environment contains many of the key environmental risk factors associated with the development of these disorders. And, because of certain behavioral differences from horses, donkeys and mules are at greater risk of their physical and mental health being overlooked. This finding, adding to the wealth of evidence for their poor health, should further strengthen efforts to replace their use in the kiln environment.

## Simple Summary

There are an estimated 60 million working donkeys and mules across the world. In countries such as Egypt, Nepal, India and Pakistan, they are used in the brick industry, carrying heavy packs or pulling carts of bricks into the kilns for firing. Their lives can be incredibly tough; charities working in these situations report problems with overworked animals, difficulties accessing water, food or shelter for them and a high frequency of untreated illnesses including dental problems, lameness and open wounds from poorly fitting harnesses or aggressive handlers. We wanted to look at the evidence that these animals may—as a result—also be suffering from poor mental health such as learned helplessness, depression or complex post-traumatic stress disorder (cPTSD). Learned helplessness is when an animal appears to ‘give up’ after being repeatedly exposed to a harmful situation from which they cannot escape. We compared the criteria and the risk factors for these disorders in humans with veterinary reports of the donkey’s behavior and living situation. We found that there was strong evidence that these animals are at high risk of suffering from one or more of these disorders and argue that because donkeys and mules do not show their feelings as obviously as horses, the severity of their situation may not be fully recognized.

## 1. Introduction

*Whilst working with the veterinarians in the Egyptian kilns, I approached this particularly thin and injured donkey and started to rub the inside of his ear. This normally provokes a strong pleasure reaction—the head droops, the bottom lip relaxes, the eyes close. But this donkey did nothing, there was no reaction. I tried scratching his withers, also something that all equines love. Nothing…no response at all…it was like he was totally shut down (Anna Harrison, veterinarian, pers obs)*

There are an estimated 50 million donkeys and 10 million mules working across the world [1]. They are used to cart goods and people, to carry water, bricks, other construction materials and waste; as draught power in agriculture; and for ceremonial purposes [2]. One of the toughest situations for these animals is working within brick kilns [3]. The task of these so-called ‘kiln donkeys’ is to transport freshly produced sun-dried bricks from the surrounding area directly into small kilns; after a sufficient quantity has been deposited, the kiln will be lit and the bricks fired. These privately owned brick kilns are found typically in Egypt, Nepal, India and Pakistan. Kiln workers and their donkeys often have a partly nomadic existence, travelling 100 km or more to the brick kiln regions for half the year [4]. Donkeys—and sometimes mules—are traditionally used as they are nimble and small enough to negotiate the narrow entrances of the brick kiln itself [4].

The animals are harnessed and either pull carts carrying the bricks or directly on their backs in packsaddles. Farhat et al. [5] found that for donkeys pulling carts in Egyptian sites, the average load of bricks was 6 to 12 times their body weight, and the average total weight of the cart and its load a metric 2.25 tons (5500 lbs). Mules in the Egyptian kilns pulled an average of 111 loads per day, with an average load weighing 765 kg (1687 lbs) (minimum 450 kg, maximum 1000 kg per load) [6]. For donkeys carrying packs in Pakistan overloading was also common, with 87% of donkeys being reported to carry more mounted load than the recommended 50% body weight ratio limit [7]. Owners estimated that the median weight of their donkeys was 110 kg (243 lbs) and that they carried a median mounted load of 82 kg (181 lbs) [7]. The animals also often negotiate difficult terrain [6] and are exposed to extreme environmental temperatures from the geography in addition to heat radiating from the ovens [5]. The condition of the carts is often questionable, with deflated tires and poor wheel bearings that make the pull even harder [5].

In addition, the animals are frequently reported to suffer wounds, sometimes severe full-depth wounds, from poorly fitting or dirty harnesses, but also from beatings from their drivers to ‘encourage’ them to move [2,5,6]. In Egyptian sites, the drivers are typically children or adolescents [6], who have insufficient knowledge, skills and attitudes to effectively communicate with their donkeys and no motivation to enhance the welfare of these equids [5]. When asked, one young boy said that “hitting them makes them run and they are happy when they run” (Broken: The brick kiln donkeys of Egypt. Safe Haven for Donkeys video, https://www.safehaven4donkeys.org/our-donkeys/videos (accessed on 21 May 2025)).

The donkey owners work under a high production pressure [5,8]. A typical working day is 8–12 h [5,6], and the donkeys may only have one rest day per week, if at all [2]. In the study of the kilns in Gujarat, Kubasiewicz et al. [8] found that only 5% of owners rotated their donkeys so that they could each have a day off. Reflecting the pressure the donkey owners were under, one said: “If two or three donkeys don’t work it’s a big problem for me…. I can’t do my full quota with the remaining donkeys.”

The high production pressure on the donkey owners contributes to handler aggression, which can be exacerbated if the animal also responds with self-protective behavior perceived as aggression, leading to a cycle of abuse. Ali et al. [6] reported that when mules were overloaded, they would show signs of aggression to which the handler responded by beating and slapping them to encourage them to move. The mules then responded with more overt aggression such as kicking back, causing the handlers to react with more force. Worryingly, Ali et al. [6] also reported that handlers would create open wounds on the bridge of the mules’ nose in the belief that this would improve their level of control over them.

During rest periods during the working day, donkeys often remain tethered or harnessed alone or in pairs [2]. Consequently, they have little opportunity during a typical day to socialize freely or engage in truly restorative resting behavior such as lying down, which may contribute to chronic fatigue [9]. Equines need at least five hours of sleep per day, some of which necessitates lying down to obtain REM sleep [10]. Contributing to this, there may be no purpose-built shelters for the donkeys, and they are often tethered in full sun with limited access to water [2,8]. Water is typically provided at set times as it must be fetched by the donkey owner; grazing areas, if there are any, are often not close to the kiln [8].

In addition, lack of proper veterinary care results in untreated lameness, wounds, eye lesions, parasite burdens and respiratory diseases; lack of dental care and insufficient fodder further contributes to malnutrition and gastrointestinal illness [2,11].

Animal welfare charities such as the Donkey Sanctuary, Safe Haven for Donkeys and the Brooke have over the years invested in on-site veterinary programs to monitor and improve the working conditions of these animals. As a result, the poor physical health of kiln donkeys and mules is now well recognized. However, there has been little analysis of the impact of this environment on their mental health. Some of the reports from this work do suggest that the animals may be depressed [5,12,13]. Anecdotes such as the one at the start of this paper would support this. The donkey’s behavior is typically described as ‘unresponsive’ [2,5], which is a behavior profile typically seen in individuals with learned helplessness [14].

Learned helplessness is a theory of behavior that was infamously demonstrated in the 1960s by US scientists. Simply put, it is a psychological condition whereby individuals ‘learn’ that they have no control over unpleasant or harmful conditions, that their actions are futile and that they are helpless. After enough experience of inescapable pain, suffering, or discomfort, individuals simply ‘give up’ trying to avoid it even when the opportunity arises to truly escape. In a series of incredibly cruel experiments, Overmier and Seligman [15] and Seligman and Maier [16] reported that the earlier exposure of dogs to unavoidable shocks interfered with their ability to learn to escape in a subsequent, similar aversive situation. No matter what the animal tried in the previous exposure, they essentially experienced that the shock was inescapable. In Maier’s own words: “the dogs do not look as though they are adapted; they howl, defecate, and urinate to the first shock presentation in the shuttle box. On later trials, the dogs are passive; but they whimper and jerk with the shock.” [17]. It was later suggested that it was not the experience of shock per se that interfered with subsequent avoidance learning, but the uncontrollability of this experience [18]. In a review of the theory 50 years later, Maier and Seligman [19] admitted that they had got it backwards. The passivity of the dogs in the shock experiments was not ‘learned’, but rather they ‘failed to learn’ because they were being exposed to a prolonged aversive situation. The neurobiological explanation was that when behavioral responses fail to result in escape from the stressor (as is the case if stress is uncontrollable), profound inhibition of dopamine release in the nucleus accumbens occurs and behavioral despair or helplessness follows [20].

The concept of learned helplessness has lost scientific interest in more recent times, perhaps because it is associated with such cruel experimental protocols, but also because the concept itself is relatively simple and only describes one symptom or negative thought behavior that might feature in more complex mental health conditions such as depression and post-traumatic stress disorder (PTSD). Indeed, at the time of his experiments, Seligman suggested that learned helplessness could be a laboratory model of clinical depression [21]. And, Maier and Seligman later reported that they had found that the animals (and humans) they had induced with learned helplessness showed, in combination, eight of the nine symptoms in the Diagnostic and Statistical Manual of Mental Disorders (DSM) for major depressive disorder: sad mood, loss of interest, weight loss, sleep problems, psychomotor problems, fatigue, feelings of worthlessness and indecisiveness or poor concentration [19]. PTSD is a mental health condition that can develop after experiencing or witnessing a traumatic event. It is characterized by persistent symptoms like intrusive memories, nightmares, flashbacks and avoidance of reminders of the event [22]. Complex PTSD is a more recent diagnosis recognizing the progression of trauma when the traumatic event is repeated or prolonged and where escape is not possible [22]. A diagnosis of cPTSD is made when the criteria for PTSD are met plus three further symptoms of disturbances in self-organization [22]. cPTSD therefore has similarities with learned helplessness, with inescapable trauma being its main causal factor.

In fact, in 2008, Hall et al. [14] made the case that ridden horses *in general* may be at risk of learned helplessness because their training and environment may include factors such as lack of control and inescapable pain. They suggested that horses that are perceived as ‘switched off’, lazy, passive or ‘bomb proof’ may in fact be exhibiting signs of learned helplessness, if not depression, and that these terms are a misunderstanding of normal horse behavior. Furiex et al. [23] found indeed that riding horses perceived as ‘withdrawn’ were less likely to eat sugar, an established test for anhedonia. Despite this key paper in 2008, there has not been much development of this theory for donkeys and other equines in general. Because of their apparent, and yet unfathomable, compliance with their workload and our observation that they also seem ‘switched off’ (see anecdote above), we wanted to explore the evidence that donkeys and mules may be exhibiting learned helplessness in the kiln environment.

For an animal condition to be deemed homologous to a human illness, it must display several forms of validity; symptoms should seem analogous to those of affected humans (face validity), and the animal’s condition should mimic the human disease in terms of risk, protective and therapeutic factors (predictive validity) [23]. Both Bradshaw et al. [24] and Ferdowsian et al. [25] used this approach to draw analogies with the symptoms of PTSD in rehabilitated chimpanzees. They compared the behavioral, risk and therapeutic factors reported in case studies of chimpanzees that had experienced trauma with the same factors in human PTSD. Here, we wanted to apply a similar approach to a different animal species: donkeys and mules. Given the similar etiology of learned helplessness to cPTSD and the similar symptomatology of learned helplessness to depression, we chose to look at all three. The aim of this study was therefore to compare the reported behavior of the animals and the risk factors present in the kiln environment with these three mental health conditions.

## 2. Materials and Methods

This study is a narrative review; a systematic review was not conducted as there was only a suspected limited number of papers, and a defined search may not have retrieved them all. An initial search using PubMed was conducted using the terms: welfare, kiln, donkey, mule. Further papers were retrieved by looking at the citation lists of these initially retrieved papers. Whilst the entire literature was used for background, for the purposes of reviewing the health and behavior of the animals, only those papers that constituted a survey of the health and/or behavior of a population of kiln donkeys and/or mules over the last 20 years were selected. Papers that covered a wider demographic (i.e., load-bearing equids) were included only if a portion of the sample included donkeys and/or mules working in kilns. Where possible, only the health and behavior results relating to the kiln donkeys and mules were retrieved. Data on horses were excluded, and there were too few surveys to facilitate a comparison of horses with donkeys and mules in the kiln environment. Papers were grouped together as a single survey if more than one paper reporting from the same survey was retrieved. Where papers reported the same health and behavioral parameters and on the same scale, the weighted average between the studies was given for ease of presentation of the results.

To assess the evidence that these animals may have learned helplessness, depression and/or cPTSD, we compared the reported behavior of the animals with the criteria for these disorders in humans. We also compared the kiln environment with the common risk factors for the induction of these disorders in so-called laboratory models of learned helplessness and depression in animals as well as clinical and epidemiological studies in humans. It was not possible to compare the neural biology or the therapeutic factors of these disorders for these animals as they have not been reported. Therefore, this review limits itself to looking at the behavioral and environmental risk factors only.

The behaviors reported in the animals in these surveys were compared against the behavioral criteria for learned helplessness, major depressive disorder and cPTSD. The criteria for learned helplessness were as described by Couto and Pilati [26], based on the work of Maier and Seligman [17] and McKean [27]. Learned helplessness is caused by exposure to inescapable pain, suffering or discomfort and presents as a depressed mood, passive behavior, appearing to ‘give up’, signs of fear and/or anxiety, feelings of frustration, a decreased problem-solving ability, procrastination and feelings of worthlessness [26]. The criteria for depression used the qualifying symptoms of major depressive disorder as listed in the Diagnostic and Statistical Manual of Mental Disorders (DSM-5-TR) [28]. These are feeling sad, weight loss or gain, sleeping problems (too much, too little), loss of interest in pleasurable activities (anhedonia), psychomotor problems (moving slowly or with agitation), feelings of fatigue, feelings of worthlessness, difficulty concentrating and suicidal thoughts [28]. The criteria for cPTSD used the criteria from the World Health Organization (WHO) International Classification of Diseases (ICD) 11th edition [22]. The definition of cPTSD starts with the basis of PTSD: exposure to an event or series of events of an extremely threatening or horrific nature, following which the patient experiences difficulties re-experiencing the traumatic event (nightmares, flashbacks), deliberately avoids reminders of the traumatic event and experiences hypervigilance or startles easily. In cPTSD, the trauma was more likely prolonged or repetitive and from which escape was difficult or impossible. It is additionally typified by disturbances in self-organization: difficulties in emotional regulation, negative self-concept, and interpersonal relationship challenges. Problems in affect regulation include heightened emotional reactivity to minor stressors, violent outbursts, reckless or self-destructive behavior, dissociative symptoms when under stress and emotional numbing, particularly the inability to experience pleasure or positive emotions. Negative self-concept is defined as persistent beliefs about oneself as diminished, defeated or worthless, accompanied by deep and pervasive feelings of shame, guilt or failure related to the stressor. Interpersonal relationship challenges are persistent difficulties in sustaining relationships and in feeling close to others. The person may consistently avoid, deride or have little interest in relationships and social engagement more generally [22].

The surveys were then reviewed for evidence of the presence of the following environmental risk factors that are used to induce learned helplessness and/or depression in laboratory studies using animals; see Table 1. Table 1 also provides references supporting a link between that factor and learned helpless and/or depression in humans. Any evidence of a specific link reported in the surveys between that risk factor and negative behavior in the animals was also recorded.

## 3. Results

### 3.1. Studies Retrieved

Seven unique surveys of the health and/or behavior of donkeys and/or mules in brick kilns from 2005 to 2025 were retrieved; see Table 2. Six surveys looked at both the health and behavior of the animals and one survey looked only at their health.

### 3.2. Comparative Behavior

Table 3 summarizes the percentage of donkeys and/or mules that were reported with health and behavioral parameters that were recorded in a similar manner between the surveys. Six surveys all reported an assessment of the general demeanor of the donkey or mule by an observer from a distance. Four used the same scale originally developed by survey 7, with categories of alert, apathetic or depressed. ‘Alert’ was defined as “Responding to surroundings e.g., ears moving and often forward, eyes open, feet may be moving, tail swishing, head up unless sniffing or eating”. ‘Apathetic’ was defined as “Passive response to surroundings e.g., small ear movements, some tail swishing, feet may be moving, eyes may be half-closed, head may be lowered”, and ‘Depressed’ was defined as “Unresponsive to surroundings, e.g., ears still and lowered, eyes closed or half-closed, no tail swishing or foot movement, head lowered”. A total of 15% of the donkeys or mules on average were classified as apathetic or depressed (range 10–82%). Only surveys 3 and 6 separately reported the proportion that were ‘depressed’ (19% and less than 1%, respectively). Surveys 1 and 2 used the Equid Assessment, Research and Scoping (EARS) tool [45] that has more categories (at ease, alert/active, aggressive/agitated and apathetic/depressed) and reported the percentage as apathetic/depressed as 10% and 2% (donkeys and mules), respectively.

Five surveys also assessed the animal’s response to an approach by an unfamiliar person in a similar way. A total of 57% of the donkeys or mules on average failed to react to an approach by an unfamiliar person (range 25–65%); see Table 3. Most of the remaining animals in the surveys showed a negative response rather than a friendly response, 33% on average (range 24 to 68%). This was defined as either turning or moving away or showing aggressive behavior such as a bite threat (roughly half performing one or the other). Survey 1 reported that 20% of donkeys showed an aggressive response, survey 4 reported that 30% of mules showed an aggressive response and survey 3 reported that 37% of donkeys showed an aggressive response. Survey 6 reported that less than 1% of either donkeys or mules showed an aggressive response.

Surveys 1 and 2 using the EARS tool also looked in more detail for signs of fear and distress when the animals were standing quietly. Survey 1 found that 54% of donkeys and survey 2 found that 25% of donkeys and 27% of mules showed signs of fear and distress, described as head shyness, unpredictable or sudden movements and sudden ‘startle’ reactions.

Table 4 lists the characteristics of learned helplessness, the qualifying symptoms of major depressive disorder and cPTSD and compares them with the descriptions of the behavior of kiln donkeys and mules from the six surveys that reported on their behavior.

There was evidence of five indicators of learned helplessness (exposure to prolonged, inescapable trauma, depression, signs of fear or anxiety and passivity, including anecdotal evidence for the general demeanor of ‘giving up’) out of seven that are assessable in animals. The anecdotal evidence comes from the author’s observations (pers obs, A.H.) and general observations from surveys 2 and 3. Two indicators were not assessed in these surveys (decreased problem-solving ability and frustration). Two indicators (feelings of worthlessness and procrastination) are not possible to assess easily in animals.

There was evidence of two indicators of depression (depression, problems with affect regulation) out of three that are assessable in these animals. One indicator was not assessed in these surveys (decreased problem-solving ability/difficulty concentrating). Six indicators of depression are not possible to assess (feelings of worthlessness, suicidal thoughts) or are impossible to separate from the effects of the kiln environment (psychomotor problems, fatigue, sleeping problems and weight loss). Many of the animals are fatigued, show weight loss and may be described as slow (necessitating to be beaten to speed up, reported in survey 4) and may have difficulty sleeping because of the restraint. So, it is impossible to determine if these indicators are the cause or effect of depression.

There was evidence of three indicators of cPTSD out of four that are assessable in animals (exposure to prolonged and inescapable trauma, enhanced startle reaction, problems with affect regulation). Two indicators were not assessed in these studies (decreased problem-solving ability and difficulty with relationships). Three indicators of cPTSD are not possible to assess (feelings of worthlessness) or are not relevant because the trauma has not ended, but regardless, they would be difficult to assess in these animals (re-experiencing the traumatic event after the traumatic event has occurred and deliberate avoidance of reminders of the traumatic event).

Two surveys reported sudden ‘startle’ reactions in the donkeys when standing quietly, which is one symptom of cPTSD (22% of donkeys—survey 1, 3% of donkeys and 1% of mules—survey 2). Difficulties in affect regulation that were consistent with the criteria for depression and cPTSD, that is difficulties in managing and responding appropriately to emotional experiences (tending to over or underreact), included unpredictable or sudden movements (distinct from startle reactions) (8% of donkeys—survey 1, 8% of donkeys and 9% of mules—survey 2). Some of the animals also showed aggression toward a non-threatening approach by an observer, which could indicate over-reactivity. Conversely, a proportion of the animals tended not to react to an observer, which could reflect dissociative symptoms. It is the author’s observation (pers obs, A.H.) that some of the donkeys show anhedonia (lack of pleasure) in their general behavior, for example by appearing to have given up and being largely unresponsive to positive human interactions. However, this was not explicitly assessed in the surveys. The evidence for the third indicator of cPTSD, difficulty with relationships, is very limited based on the surveys. Failure to react to, or aggression towards, human observers could be relevant to the third indicator of cPTSD—difficulty with relationships—but the observer was unfamiliar and not a conspecific. Anecdotally, the donkeys can be aggressive to each other, particularly over resources (pers obs, A.H.), but it is fair to say that conspecific relationships have not been assessed in these surveys.

### 3.3. Comparative Risk Factors

There was evidence for the presence in the kiln environment of all six environmental risk factors used to induce learned helplessness and/or depression in laboratory animal ‘models’ and also associated with these disorders in humans:

- Restraint

Donkeys and mules are typically worked for between 6 and 12 h per day [2,5,6] and 6–7 days per week [2,8], during which time they are either harnessed into carts or carry packs with a human driver. During rest breaks when working, the harness typically remains on them [2]. So, they are effectively restrained whilst working. After working, they are usually tethered [2,8] or hobbled [4]. Hobbling is a method of restraint to prevent the animal moving away; usually, two of the limbs are tied together towards the base of the leg or tied to an immovable object using a chain or tight rope. When unsuitable or dirty materials are used to hobble or tether, it tends to cause characteristic hair loss and lesions. A total of 32% of the donkeys and 43% of the mules had signs of limb tethering (rope burn type lesions) in survey 2, and 89% of donkeys and 79% of mules had hobble/tether scars in survey 7.

- Abuse/Coercive control

Beating by owners to make the donkeys speed up was observed in 43% of the kilns in survey 1. A total of 65% of handlers in survey 4 reported using nose ropes/metal chain for driving their mules, and 67% claimed that mules must be beaten to work properly. Mistreatment-induced lesions (42% of mules) were more predominant in survey 4 than other categories of body lesions. A total of 49% of donkeys (survey 3) and 12% of donkeys and 6% of mules (survey 7) had a beating wound on their hindquarters caused by drivers repeatedly hitting the donkeys in the same area. However, whilst 98% of the donkeys in survey 1 had scars, alopecia, swellings or scabs, see Table 3, with 42% having open wounds at the time of assessment, these were predominantly due to bites from other donkeys and poorly fitting harnesses (survey 1).

The following associations between aggressive behavior by the handlers and negative behavior in the animals were reported. Donkeys were more likely to respond negatively (fear/aggression) to an approach by an unfamiliar person if they had been roughly handled by their owner (survey 1). Mules were more likely to be aggressive towards an unfamiliar person if they were made to work longer or with heavier loads or had had a younger, less experienced handler or one with a belief that mules were difficult to handle (survey 4). Mules that exhibited aggressive or avoidance behavior toward an observer were more likely to suffer from mistreatment-induced lesions (survey 4). Equids with aggressive handlers were more likely to avoid or be aggressive to an observer or show agitated, hyper-reactive, hyper-vigilant or fearful behavior when standing quietly (survey 2).

- Chronic pain

Chronic pain, mostly from harness or beating-induced body lesions or from lameness, appears to be ubiquitous in the kiln environment. A total of 53% of donkeys and mules were reported with scars and/or open wounds (range 37–98%); see Table 3. Survey 1 reported that 98% had scars, alopecia, swellings or scabs, with 42% having open wounds from bites from other donkeys, poorly fitting harnesses, or beatings. Survey 3 reported that 80% of the donkeys had some type of wound; 49% had beating wounds, which were caused by drivers hitting the donkeys (mean size 48 cm^2^); 43% had a saddle wound (mean size 26 cm^2^); and 40% had a neck collar wound (mean size 23 cm^2^). Survey 4 reported that 42% of mules had mistreatment-induced lesions, 25% had harness lesions, 20% had overwork-induced lesions and 28% had deep lesions. Survey 7 reported that 18% of donkeys (28% of mules) had lesions on their girth, 12% (6% of mules) on their hindquarters (probably from stick beating during work), 11.5% on their breast/shoulder (23% of mules) and 10% on their withers (21% of mules).

Reports of hoof problems and lameness varied in their detail of reporting, preventing a weighted average being determined, but prevalences were very high in general. Survey 6 reported that hoof and limb problems were over 90% prevalent across all the equids in their study. Survey 7 reported that 92% of donkeys had limb deformities (77% of mules), and 95% had gait abnormalities (91% of mules). Survey 1 reported that 49% of donkeys had overgrown hooves and 28% had hooves which were chipped or cracked. Another study that focused on the donkey owners reported that at some point in their donkey’s life, 65% had seen load-associated injuries, of which 28% were wounds, 21% were lameness and 7% were back pain [46]. In a parallel study, 42% of donkey owners reported seeing lameness while their donkey was working [7]. Furthermore, survey 7 reported that 77% of donkeys had tooth problems (85% of mules).

The following associations were reported; apathetic animals were more likely to have more severe wounds (survey 6), and across all the equines, there was a correlation between lack of response to an unfamiliar person and low body condition score, body lesions and gait problems (survey 7).

- Chronic stress

Chronic stress from the workload, restraint, chronic pain and beatings is exemplified in the preceding text. Additional stress on the body caused by food, water and sleep deprivation was also common. Survey 7 reported that 37% of donkeys and 46% of mules had signs of dehydration, and survey 1 found that only 19% of donkeys had a ‘good’ general health score. Body condition score (BCS) is a commonly used scale in equine welfare to measure healthy weight; 5 = very fat, 4 = fat, 3 = good, 2 = thin, 1 = very thin. Across six studies, 62% of the donkeys and mules on average had a body condition score of thin or very thin (range 42–96%); see Table 3. Associations between poor body condition were found with negative behavior in the donkeys and mules. Survey 3 found that donkeys with a higher BCS showed more alert behaviors and were friendlier with humans. Survey 4 found that mules were more likely to be aggressive towards an unfamiliar person if they had a low BCS, were made to work longer or with heavier loads or had a younger, less experienced handler or one with a belief that mules were difficult to handle. Survey 6 found that animals classified as ‘apathetic’ were more likely to have a low BCS, abnormal mucous membrane color, fecal soiling, eye abnormalities, more severe wounds, and older age. In survey 7, across all the equines, there was a correlation between lack of response to an unfamiliar person and low BCS, body lesions and gait problems.

- Lack of control

The entire kiln environment is completely inconsistent with any control for the animal due to being forced to work, persistent restraint and no freedom to express normal behavior, including even the ability to seek water or food even when resting [9].

- Unpredictability

Witness reports detail that the animals can frequently receive conflicting instructions from their drivers (to speed up/slow down) [47], and this is exacerbated if the driver/rider is young and inexperienced [5].

Mules were more likely to be aggressive towards an unfamiliar person if they had a younger, less experienced handler or one with a belief that mules were difficult to handle (survey 4).

There was evidence of associations with indicators of chronic pain (such as wounds and lameness), chronic stress (such as low body condition score) and abuse (beatings or aggressive owner) and depressed behavior in the donkeys.

Whilst aggressive behavior was generally low in the donkeys and mules (see above), where it was looked at, there was also evidence of associations with abuse (beatings or aggressive owner), chronic stress (low body condition score) and lack of predictability (young drivers) with aggressive behavior by the donkeys/mules towards an unfamiliar person. It is notable that in the survey 2 study, where the handlers were generally not reported to be aggressive to their donkeys, there were low reports of depression (2%) and aggression (2%) in the donkeys. However, the authors did still see a correlation between aggressive handlers and fearful or aggressive behavior in those donkeys. Aggression to observers was considered by the authors of survey 4 to be fear-related.

## 4. Discussion

### 4.1. Learned Helplessness, Depression and/or Complex Post-Traumatic Stress Disorder?

We found seven large-scale surveys of the health and behavior of donkeys and mules working in the brick kiln environment over the last 20 years, all funded by equine charities. They consistently reported that a proportion of the animals presented as apathetic or depressed (15% on average, although the range is large, 10–82%). Furthermore, a large proportion of the animals failed to respond—at all—to an approach by an observer (57%, range 25–65%). Those that did respond tended to respond negatively by showing avoidance or aggression (33%, range 24–68%), probably indicative of fear [6]. A significant proportion of the donkeys in two studies (54% survey 1, 25%, survey 2) were reported to show unprovoked signs of fear and anxiety including startle reactions and sudden movements, which may be indicative of poor affect regulation.

Using these seven quantitative reports, we used a comparative approach to assess the evidence that a proportion of these animals may have learned helplessness, depression and/or cPTSD based on face validity (comparable behavior) and predictive validity (comparable risk factors). The limited quantitative behavioral assessment by veterinarians or trained assessors on behalf of the equine charities did, in our view, support a behavior profile consistent with learned helplessness and/or cPTSD. There was evidence of five indicators of learned helplessness (exposure to prolonged, inescapable trauma, depression, signs of fear or anxiety, passivity and the general anecdote of ‘giving up’) out of seven that are assessable in animals. And there was evidence of three indicators of cPTSD out of four that are assessable in animals (exposure to prolonged and inescapable trauma, enhanced startle reaction and problems with affect regulation). Partly due to the criteria for cPTSD, which involved not only behaviors that had been assessed but also causal indicators, it seems like this may more closely reflect the donkey and mules’ experience, albeit that the trauma is not ‘post’, i.e. it has not ended. It is also notable that the base cause of cPTSD—exposure to prolonged and inescapable trauma—is the defining cause of learned helplessness.

Using the human-based criteria for depression seemed to be less useful to characterize the donkey and mules’ behavior, aside from the general assessment of appearing ‘depressed’. Unfortunately, six of the nine criteria for depression listed under the DSM-5-TR are not possible to assess in animals or are impossible to separate from the effects of the kiln environment itself. Fatigue, weight loss and sleep problems seen in the kiln donkeys are more likely causal of poor mental health—chronic stress is, after all, linked to depression [38]—rather than a symptom, although it is impossible to know.

The kiln environment is full of the same stressors that are used to create ‘animal models’ of learned helplessness and depression in the laboratory. These are also well-known risk factors for the development of learned helplessness, depression and cPTSD in humans. Restraint, coercive control/abuse, lack of control for the animals, chronic pain and stress are ubiquitous for the donkeys and mules in the kilns. A total of 53% of donkeys and mules were reported with scars and/or open wounds (range 37–98%), and 62% on average had a body condition score of thin or very thin (range 42–96%). There was evidence that the lower the pain and stress levels in the animals, the more likely they were to appear alert and friendly.

Other studies on a wider range of working equines have also reported a link between depressed behavior and chronic pain and stress. A survey of 5000 working equines found a correlation between apathetic/depressed animals and incidences of chronic pain, dehydration and heat stress [48]. Correlation was also found between the depressed attitude of working horses and the presence of deep body lesions [49].

Stereotypic behavior in animals is the conduct of seemingly pointless, repetitive movements such as circling, weaving and pacing and is a known coping response to stressful, typically impoverished, environments [50]. Mules, and more so donkeys, are reported to be less likely to engage in stereotypies than horses [51]. In working equids, an alternative coping mechanism to stereotypies could be a form of apathy and learned helplessness [14]. ‘Being awake but motionless’ has been suggested as a reaction to non-enriched housing that is alternative to stereotypic behavior and could reflect depression-like states [52]. This reaction may be more likely if the animals are already exhausted and do not have the energy for stereotypic behavior. Indeed, survey 2 suggested that the apparent calmness of the equines in their study could be a behavioral manifestation of learned helplessness or hopelessness.

The reports of a link between these factors and aggressive reactions to observers does not necessarily mean that the donkeys and mules were aggressive (in fact, between 1 and 37% were reported to be) but simply that that was a factor that had been looked at, most notably by survey 2 and 4. Survey 4 concluded that the aggression was most likely fear-based because it was linked to mistreatment. Nonetheless, the fact that a proportion of the animals did respond somewhat aggressively could lean more to a diagnosis of cPTSD rather than learned helplessness for those animals.

### 4.2. Why Has Their Mental Health Been Overlooked?

Whilst there has been a lot of effort in defining and assessing the welfare of kiln donkeys in the last 20 years, it has been performed with a view to determine how best to improve their situation and how to measure the impact of these interventions, mainly with respect to their physical health. Several of the authors of the surveys reported that they felt the animals were indeed depressed [5,12] and even mentioned learned helplessness as an explanation for their apathy and apparent compliance with their situation [2,5]. However, given the overwhelming evidence for the poor physical health of these animals (thin body condition, wounds and lameness to name a few), their mental health has remained somewhat of a side issue.

Another reason why learned helplessness and depression in working donkeys and mules has been overlooked is that they are different to horses. The donkey evolved from the African wild ass that inhabited mountainous, semi-arid regions, in contrast to the open, grassy plains of the ancestral home of its cousin the horse. It was the first equid to be domesticated more than 5000 years ago. This domestication was likely because of its gentle nature and its lower tendency to flee like horses and other equids when under threat [51]. As donkeys do not tend to live in herds like horses, fleeing is not the best defense mechanism as you, or your foal, will still likely be attacked. Furthermore, the mountainous terrain in which they tend to live is not conducive to flight [53]. It is said that donkeys may therefore be more likely to engage in the ‘fight’ response [53]; however, studies of working donkeys have shown that they are still less likely to show signs of aggression towards an unfamiliar person compared with mules or horses [11,54]. This gentle nature combined with a tendency to freeze or react cautiously to threats has led to the belief that donkeys, and to some extent their mule hybrids, are stoical [53]. Donkeys also respond more subtly to pain or discomfort than other equids, further reinforcing the stoical stereotype [9,53]. The perception of their apparent stoicism and higher tolerance to pain has been recently dismissed as a misunderstanding of donkey behavior [9,47,53,54]. The belief that donkeys do not feel pain may be due to people looking at donkeys and judging them using the wrong behavioral and physical scales, most notably those of the horse [9]. Disguising of pain may be a mechanism to prevent predatory attacks, which is common in prey animals [54]. Because of this, the physical and mental welfare of donkeys and mules can more easily be overlooked [9,53,55]. Symptoms of learned helplessness or depression may be ignored and falsely construed as normal donkey demeanor.

Unfortunately, this stoical nature and tendency to react cautiously rather than flee like a horse has also led many to consider donkeys as stubborn [9,51,56], leading them to be treated even more harshly [6,54]. Indeed, survey 7 found that donkeys had higher prevalences of gait abnormalities, eye abnormalities, and hindquarter lesions (potentially from beating or cart injuries) than horses or mules. And survey 5 found that donkeys were more at risk of body lesions than mules.

Donkeys and mules are also perceived to be more robust than horses; indeed, they are relatively tolerant of droughts and poor-quality feed (53,57] and may be less thin than horses under similar conditions [44]. The belief that donkeys and mules are more robust than horses contributes to a continuation of under-nutrition, inadequate hydration, and in most cases, insufficient overall welfare conditions [57]. In fact, donkeys, with enhanced nutritional demands when working for humans, rather than when roaming free in a semi-arid area, are very difficult to feed and keep at good health [58]. Donkeys are also typically less expensive than horses and tend to be owned by people who are in extreme economic and societal hardship, so they may be more likely to be overworked and have lower-quality husbandry than horses [3].

Add the lower tendency to flee or fight and a masking of signs of pain and distress to an animal that is able to survive on a poorer diet and you have an animal that is in a particularly vulnerable position in the brick kiln environment, where socio-economic pressures may take precedence [8].

### 4.3. Study Limitations

There was a consistency to the behavioral assessment made by the six surveys, which makes comparison with the studies much easier. However, the behavior assessment was still rather rudimentary; limited to an assessment of the animal from a distance (alert, apathetic, depressed) and their reaction to the approach of an unfamiliar person (interested, ignore, avoid, show aggression). Therefore, it was only possible to classify the animals as alert, non-alert (apathetic/depressed), aggressive, non-responsive or interested/friendly. We have made some assumptions that ‘non-reactive’ animals fit the criteria for passivity (a criteria for learned helplessness) but also that they might fit the criteria for problems with affect regulation seen in cPTSD. We also assumed that aggressive responses to observers may also fit the criteria for problems with affect regulation seen in cPTSD. Whilst the behavior of the donkeys, at least to the extent it was assessed, fitted more of the behaviors for learned helplessness, there was some evidence that a diagnosis of cPTSD might also be applicable. However, to be sure, more nuanced information is needed, which would probably entail a more experimental approach, for example by assessing the animal’s reaction to more specific events, including interactions with conspecifics and by giving them choices. In particular, a more rigorous assessment of lack of interest or pleasure (anhedonia) is needed (see [23]) as we are relying here on personal observation.

Currently, we simply do not know whether the animals have learned helplessness because most of the time they do not have opportunity to escape their situation. To test this, one could relatively easily perform an in-the-field experiment, giving the donkeys the genuine option to walk out of the kiln environment. The fact that the animals are frequently hobbled or tethered when not working [2,4,8] might suggest, however, that they *would* choose to leave if given the choice and therefore may *not* have learned helplessness. Again, this might suggest that a diagnosis of cPTSD for some of the animals is more appropriate.

Some of the surveys also concluded that the animals were depressed (survey 3 and 6), but they, and others, also highlighted that this unresponsive behavior profile could also be consistent with sickness behavior, exhaustion or chronic pain [5,12,13,47]. The animals’ resources are being stretched to their limits, and they are conserving previous energy by failing to respond to stimuli that would normally be considered threatening [12]. The irony is that in failing to refuse to undertake tasks because of this apathy, they are at risk of being pushed to even more extreme limits of their physical health [47].

Indeed, it is currently impossible to determine if these animals are traumatized or depressed or just exhausted and sick. Chronic pain and stress, however, are strongly linked to depression and learned helplessness in humans and laboratory studies in animals, so it is perhaps reasonable to assume that the donkeys and mules are also probably depressed.

Not all the animals in each survey were reported to be depressed or fearful. However, this does not imply that the welfare of the rest of the group is good. Indeed, in relation to other indicators of poor welfare such as stereotypies, the consensus is that if a proportion of the animals appear to be struggling, then that indicates that the situation is poor for the welfare of all [50].

### 4.4. What Can Be Done?

The fact that donkeys keep their owners from extreme levels of poverty, however, has the unavoidable outcome that owners may not be able to afford to care for them adequately, despite possible aspirations to do so: “Our intention is to keep them [for a long time], but we don’t have enough food, money and other resources to keep them well” [8]. These aspirations are also easily eroded as the pressures of poverty result in a sense of powerlessness to affect change [43]. Indeed, the people owning the donkeys are often in a form of servitude to the kiln owners, owing them money to buy their donkeys, and they are also in extreme poverty [8]. So, in a way, the donkey owners themselves may be suffering from learned helplessness as a consequence of their situation, perpetuating the cycle of abuse.

Kubasiewicz et al. [8] argue that the mental and physical conditions of the donkeys mirrored that of their owners, and this shared suffering between humans and their donkeys highlights the need for a ‘one welfare’ approach to intervention work aimed at improving the health and welfare of both donkeys and humans. They found that donkey owners that were better off financially tended to have donkeys with better welfare [43]. So, improving the financial situation for the donkey owners is one solution, but it may not be sufficient to bring their situation from one of ‘dire’ to ‘good’.

Given the prevalence and relative inexpensiveness of donkeys, there has been little motivation to change to more modern technologies/machinery. In poor countries, donkeys and mules are still indispensable as cheap and robust load carriers, especially in hot and arid climates and in places where transport infrastructure is insufficient [56]. The sea change will likely come from the economic advantage of brick production using more efficient machinery and therefore increasing profits rather than from deep concerns over the welfare of animals used [59]. The move to modern machinery also enhances the ‘one welfare’ approach as the young boys working at the kilns will have the advantage of learning to drive and maintain modern machine equipment, a life skill that could be one route out of their disadvantaged position. Some believe that mechanization of the Egyptian brick kilns may not be realistic without entirely renovating all kilns for additional space requirements, which would be cost-prohibitive [11]. For others, the solution for the donkeys, their handlers and the kiln owners lies in developing an ideally configured tractor, which would circumvent the need to restructure the kilns. Even further, new initiatives using modern brick-making machines imported from China could also be the start of the revolution, freeing both donkeys and their owners from slavery and realizing even higher profits for the kiln owners (https://www.eurasiareview.com/04042025-breaking-the-cycle-mechanization-brings-hope-to-pakistans-brick-kiln-workers-oped (accessed on 21 May 2025)). A win–win solution is feasible once the will is there.

## 5. Conclusions

Using seven quantitative veterinary surveys, we used a comparative approach to assess the evidence that a proportion of these animals may have learned helplessness, depression and/or cPTSD based on face validity (comparable behavior) and predictive validity (comparable risk factors). The limited quantitative behavioral assessment did, in our view, support a behavior profile consistent with learned helplessness and/or cPTSD. There was evidence of five indicators of learned helplessness out of seven that are assessable in animals and evidence of three indicators of cPTSD out of four that are assessable in animals. Furthermore, the kiln environment had the same environmental risk factors as those used to induce learned helplessness and/or depression in laboratory animal ‘models’: restraint, coercive control/abuse, chronic stress, chronic pain, lack of control and unpredictability. These factors also have a strong association with the development of learned helplessness, depression and/or cPTSD in humans. The implication of the finding that these animals may therefore be at high risk of learned helplessness, depression and/or cPTSD adds to the already overwhelming evidence of their otherwise poor health and welfare. If these animals internally feel defeated and hopeless, they are in a dire situation. Furthermore, because donkeys and mules are stereotypically perceived as being stoical, the fact that they may have learned helplessness or depression may be being overlooked, and these types of equines may be at even greater risk of being mistreated. The findings here should further encourage efforts to help these animals out of these situations; especially after 20 years of trying, where it has proved to be impossible to raise their welfare standards to acceptable levels.

## Figures and Tables

**Table 1:** Environmental risk factors for the creation of learned helplessness and/or depression in animals and humans.

Risk Factor	Laboratory ‘Model’ of Learned Helplessness/Depression in Animals	Link with Learned Helplessness/Depression in Humans
Restraint	**Chronic restraint stress model**—rats are placed in individual transparent tubes for 2.5 h daily over 13 days [29]	Restraint and isolation (such as in a room) are well-known coercive control measures linked to major depressive disorder and other psychiatric problems [30]
Abuse/Coercive control	**Learned helplessness model**—a mouse is exposed to unpredictable and inescapable electric footshocks for two consecutive days [31] **Chronic social stress/social defeat model**—a mouse is exposed to a larger and aggressive mouse for 5 min a day and then housed across a transparent barrier to sustain sensory contact, for 10 days [31]	Abuse including psychological and physical abuse is well linked to depression [32] Learned helplessness is linked with PTSD and depression in battered women [33]
Chronic pain	**Physical pain model**—a spared nerve injury (that damages two branches of the sciatic nerve) is surgically inflicted in rats, resulting in persistent neuropathic pain [31]	Strong links between learned helplessness and patients with long-term illnesses associated with chronic pain such as fibromyalgia and rheumatoid arthritis [34] Chronic pain patients with PTSD were more likely to have learned helplessness and depression [35] Pain induces depression and influences its progression [36]
Chronic stress	**Chronic social stress/Social defeat model** **Chronic mild stress model**—mice are subjected to unpredictable mild psychosocial stressors for 9 weeks such as cage tilting, damp sawdust, no bedding, light/dark changes, food and water deprivation, predator odor, etc. [31] **Sleep deprivation model**—the light/dark cycle of mice is altered to induce wakefulness for 7 days [37]	Chronic life stress closely linked to depression [38] Poverty-related stress was associated with learned helplessness in children (in relation to education and learning) [39] Sleep problems closely linked to depression [40]
Lack of control	**Learned helplessness model**	Lack of perceived control has been associated with learned helplessness and stress-related disorders, such as depression and anxiety [41]
Unpredictability	**Chronic mild stress model** **Learned helplessness model**	Unpredictability linked to depression, particularly if experienced during childhood [42]

**Table 2:** Surveys retrieved of kiln donkeys and/or mules and whether they assessed their health and/or behavior.

Survey No.	Reference(s)	Survey Details (Field Work Year)	Health	Behavior
1	[4,8,43]	220 donkeys from 14 kilns in Gujarat, India (2018)	yes	yes
2	[2]	2448 equids from 41 kilns in Nepal (42% mules, 2% donkeys) (2019)	yes	yes
3	[5]	179 donkeys from 20 kilns in Egypt (2017)	yes	yes
4	[6]	374 mules from 50 kilns in Egypt (2016)	yes	yes
5	[11]	1140 donkeys from 185 kilns in Egypt (2013)	yes	no
6	[3,12]	5481 donkeys, 4504 horses and 858 mules across nine developing countries (including kilns) (2003–2007)	yes	yes
7	[44]	4903 horses, donkeys and mules used for draught, pack and ridden work in Afghanistan, Egypt, India, Jordan and Pakistan (including kilns) (2003)	yes	yes

**Table 3:** The percentage of donkeys and mules in the reviewed studies that reported on the same health and behavior in a similar manner. BCS = body condition score, where 2 equates to thin. * Assessed, but not on the same scale as others.

Survey No.	Donkeys, N	Mules, N	% Apathetic/ Depressed	% Non-Responsive to Observer	% Negative Response to Observer	% with BCS 2 or Less	% with Scars/Wounds
1	220		*	not given	60	72	98
2	55		*			60	37
2		1028	*			42	40
3	179		82	25	68	56	80
4		345	33	29	62	96	*
5	1140					50	
6	5481		13	65	26	*	
6		858	13		24	*	
7	2596		12	44	44	70	*
7		222	10	54	30	77	*
Weighted Average			15	57	33	62	53

**Table 4:** The characteristics of learned helplessness, the qualifying symptoms of major depressive disorder and cPTSD and the descriptions of the behavior of kiln donkeys and mules from the surveys that reported on their behavior (see Table 2 for survey source).

Sign	Evidence in Kiln Donkeys/Mules	Learned Helplessness [26]	Major Depressive Disorder [28]	Complex Post-Traumatic Stress Disorder [22]
Depressed mood	Yes (1, 3, 4, 6, 7)	yes	yes	-
Passivity	Yes, non-reaction to observer (3, 4, 6, 7)	yes	-	-
Giving up	Anecdotal reports (2, 3, pers obs, A.H.)	yes	-	-
Fear/Anxiety	Yes, avoidance or aggression to observer (1, 2, 3, 4, 6, 7)	yes	-	-
Frustration	Not assessed	yes	-	-
Decreased problem-solving ability	Not assessed	yes	Yes (difficulty concentrating)	-
Procrastination	Not possible to assess	yes	-	-
Weight loss/gain	Not possible to separate from environment	-	yes	-
Sleeping problems	Not possible to separate from environment	-	yes	-
Psychomotor problems (agitated/slow)	Not possible to separate from environment	-	yes	-
Fatigue	Not possible to separate from environment	-	yes	-
Suicidal thoughts	Not possible to assess	-	yes	-
Exposure to an event or series of events of threatening nature, most commonly prolonged or repetitive events from which escape is difficult or impossible	Yes (1–7)	yes	-	yes
Re-experiencing the traumatic event after it has occurred	Not applicable/Not possible to assess	-	-	yes
Deliberate avoidance of reminders of the traumatic event	Not applicable/Not possible to assess	-	-	yes
Hypervigilance or an enhanced startle reaction	Yes (1, 2)	-	-	yes
Problems in affect regulation (heightened emotional reactivity, dissociative symptoms, emotional numbing anhedonia)	Yes, reactivity: Sudden movements (1, 2) Aggression to observers (1, 3, 4, 7) Yes, numbing: Non-reaction to observer (3, 4, 6, 7) Anecdotal reports of dissociative behavior, anhedonia (pers obs, A.H.)	-	Yes (anhedonia)	yes
Feeling worthless, shame or guilt	Not possible to assess	yes	yes	yes
Persistent difficulties in sustaining relationships and in feeling close to others	Not assessed	-	-	yes

## Data Availability

The original contributions presented in this study are included in the article. Further inquiries can be directed to the corresponding author.
